# ACMG/AMP variant classification framework in arginase 1 deficiency: Implications for birth prevalence estimates and diagnostics

**DOI:** 10.1016/j.gimo.2024.101815

**Published:** 2024-01-23

**Authors:** Jessie M. Cameron, Mayowa Azeez Osundiji, Rory J. Olson, Bukola A. Olarewaju, Andreas Schulze

**Affiliations:** 1 Department of Paediatric Laboratory Medicine, The Hospital for Sick Children, Toronto, ON, Canada; 2 Department of Laboratory Medicine and Pathobiology, University of Toronto, Toronto, ON, Canada; 3 Department of Clinical Genomics, Mayo Clinic, Rochester, MN; 4 Genetics and Genome Biology, The Hospital for Sick Children, Toronto, ON, Canada; 5 School of Science and Engineering, University of Dundee, Dundee, United Kingdom; 6 Department of Pediatrics, University of Toronto, Toronto, ON, Canada; 7 Department of Biochemistry, University of Toronto, Toronto, ON, Canada

**Keywords:** ARG1 gene, Arginase deficiency, Birth prevalence, Hyperargininemia, Variant classification, Variant pathogenicity

## Abstract

**Purpose:**

Arginase 1 (ARG1) deficiency manifests with hyperargininemia and progressive neurological impairment. Recent estimates of birth prevalence using allele frequencies of *ARG1* variants do not sufficiently distinguish benign from pathogenic variants. Additionally, ongoing discussions of reproductive carrier screening for diseases such as ARG1 creates a need for improved understanding of *ARG1* variant classification. Here, we incorporate American College of Medical Genetics and Genomics/Association for Molecular Pathology–developed guidelines for interpreting gene variants and in silico predictions to select allele frequencies for estimation of global birth prevalence of ARG1 deficiency.

**Methods:**

We interrogated Genome Aggregation Database and PubMed for published (defined as identified in patients with clinically defined arginase deficiency in scientific literature, *n* = 73) and unpublished *ARG1* variants (defined as variants present in Genome Aggregation Database, unique to *ARG1*, but not yet associated with clinical arginase deficiency, *n* = 302). American College of Medical Genetics and Genomics/Association for Molecular Pathology guidelines were applied to classify variants using Franklin Genoox artificial intelligence–powered platform and manual review.

**Results:**

Of 73 published *ARG1* variants, 16 classified as pathogenic, 30 as likely pathogenic, and 27 as variant of uncertain significance. Of 302 unpublished *ARG1* variants, 3 classified as pathogenic, 28 likely pathogenic, and 229 variant of uncertain significance. Mutant allele frequency estimates ranged from 17 to 266 per 100,000 and birth prevalence from 1 in 141,331 to 34,602,076.

**Conclusion:**

We show that a large proportion of *ARG1* variants lack adequate evidence of pathogenicity. These findings underscore the significance of functional studies and accumulating clinical data for determination of variant pathogenicity and for improved understanding of global birth prevalence of ARG1 deficiency.

## Introduction

Arginase 1 (ARG1) deficiency (MIM 207800) is a rare autosomal recessive inborn error of metabolism that is caused by impaired L-arginine-urea-hydrolase ([EC 3.5.3.1] ARG1) function in the liver.^1^^,^^2^ ARG1 catalyzes the final step of the urea cycle, which involves the hydrolysis of L-arginine to L-ornithine and urea. The urea cycle is pivotal for nitrogen detoxification. Urea is excreted through the kidneys, whereas ornithine is returned to the mitochondria.^3^ ARG1 deficiency often manifest during preschool age with progressive loss of psychomotor functions, spastic tetraplegia, seizures, growth retardation, severe hepatic diseases, and hepatocellular carcinoma.^4^, ^5^, ^6^, ^7^ The incidence (birth prevalence) of ARG1 deficiency continues to be a subject of growing interest.^8^ Initial estimates of ARG1 deficiency birth prevalence were based on newborn screening (NBS) data and calculated to be around 1 case per million live births.^8^^,^^9^ NBS data are limited by variability of arginine levels in neonatal period and a lack of universal assessment of newborn circulating arginine levels.^10^ Considering the limitations of analysis of circulating arginine levels, potential opportunities for molecular genetic approaches to complement biochemical diagnostics for ARG1 deficiency have become increasingly necessary.

A recent study incorporated allele frequencies of published *ARG1* variants from genetic databases into the Hardy-Weinberg equation, correcting for the estimated consanguinity rate in some populations. The authors demonstrated that the birth prevalence of ARG1 deficiency was around 2.8 cases per million.^11^ One major challenge associated with integrating allele frequencies into birth prevalence calculations is accurately distinguishing benign from pathogenic variants. ARG1 gene encompasses a 15-kb genomic region with 8 exons on chromosome 6q23.^12^ The human ARG1 mRNA sequence is about 16 kb long,^13^ whereas the protein product contains about 322 amino acids.^14^ With the growing implementation of next-generation sequencing technologies, *ARG1* variants are increasingly being generated in genetic population databases. The American College of Medical Genetics and Genomics (ACMG) and the Association for Molecular Pathology (AMP) developed guidelines for interpreting genetic variants identified in Mendelian disorders about 7 years ago that is overwhelmingly being used by clinical laboratories in the United States and increasingly being adopted in many other countries.^15^ The ACMG/AMP guidelines provide guidance for classifying gene variants into 5 categories, namely pathogenic (P), likely pathogenic (LP), variant of uncertain significance (VUS), likely benign (LB), and benign (B) based on multiple types and levels of evidence such as computational data, functional data, phenotype, family history data, and population data. The guidelines are being refined^16^, ^17^, ^18^, ^19^ and may possibly be customized for some monogenic disorders,^20^^,^^21^ including inborn error of metabolism,^22^ especially with the multitude of ongoing efforts from Clinical Genome Resource (ClinGen) expert panels. In this study, we used the ACMG/AMP guidelines to classify *ARG1* variants and incorporated the variant classifications into estimation of ARG1 deficiency birth prevalence.

## Materials and Methods

### *ARG1* variants

We selected *ARG1* variants from Homo sapiens ARG1 transcript variant 1 (NM_001244438.1, NM_001244438.2), 2 (NM_000045.3 and NM_000045.4), and 3 (NM_001369020.1). This encompassed the Matched Annotation from National Center for Biotechnology Information and EMBL-EBI (MANE) select transcript.^23^ *ARG1* variants were extracted from the Genome Aggregation Database (gnomAD v3.1.2,^24^ accessed September 2021, and gnomAD v3.1.2 v2.1.1,^25^ accessed June 2022) and from the clinical scientific literature (PubMed, accessed March 2022) onto Microsoft Excel files.

The gnomAD v2.1.1 data set contains data from 125,748 exomes and 15,708 genomes, all mapped to the GRCh37/hg19 reference sequence. The gnomAD v3.1.2 data set contains 76,156 genomes (and no exomes), all mapped to the GRCh38 reference sequence. Most of the genomes from v2 are included in v3.1.

In a few cases (gross deletions^1^ and complex rearrangements^26^ involving *ARG1*) where the reported variant(s) in the clinical scientific literature did not reference a specific sequence, attempts were made to map the coordinates using University of California Santa Cruz genome browser^27^ and to validate the variants with putative protein annotations through the “VariantValidator” tool^28^ and Franklin Genoox (https://franklin.genoox.com/).

### ACMG/AMP classification of *ARG1* variants

The ARG1 gene variants were uploaded onto Franklin Genoox,^29^ an automated artificial intelligence–powered platform for ACMG/AMP variant classification. PM2 and PP5 were excluded from the evidence criteria for pathogenicity in this study.

**Figure 1:**
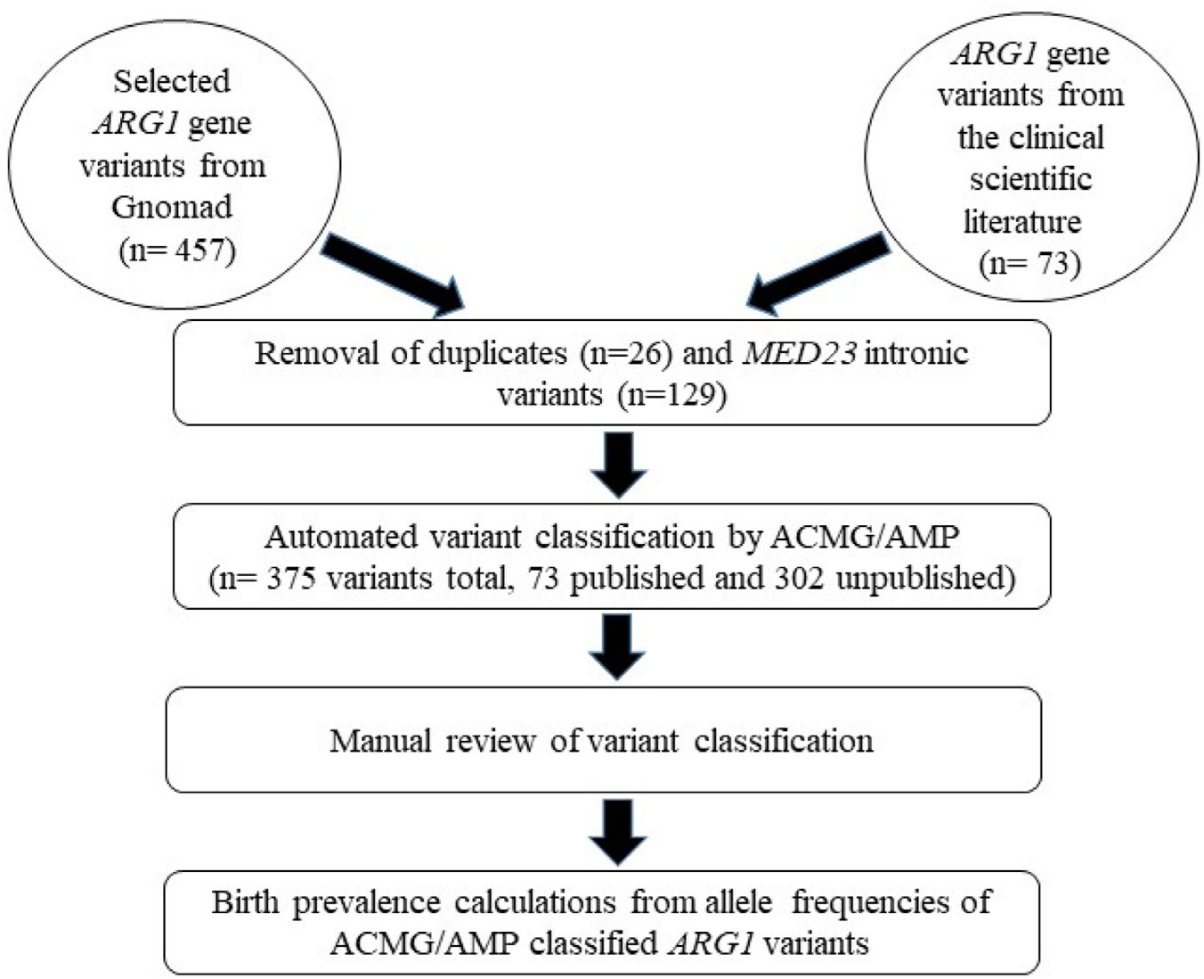
**A flowchart showing the steps involved in the analysis of *ARG1* variants by American College of Medical Genetics and Genomics/Association for Molecular Pathology** (**ACMG/AMP) variant classification framework and the estimation of ARG1 deficiency birth prevalence (models E, F, and G).** The locus of mediator complex subunit 23 (*MED23*) overlaps with that of *ARG1*.^30^

A total of 457 *ARG1* variants from gnomAD (v3.1.2) and 73 *ARG1* variants from PubMed were analyzed. We identified and removed 26 duplicate *ARG1* variants that were already present in the PubMed list. Of the remaining 436 variants, 129 intronic variants mapped to the locus of mediator complex subunit 23 (*MED23*). The *MED23* region overlaps with that of *ARG1*.^30^ Accordingly, the intronic variants (*n* = 129) overlapping with MED23 region were excluded. We studied 302 unpublished *ARG1* variants from gnomAD and 73 published *ARG1* variants. We applied ACMG/AMP variant classification^15^^,^^16^ using Franklin Genoox,^29^ followed by a detailed manual review of the medical literature. Recently suggested modifications to the ACMG/AMP variant classification criteria were applied.^16^^,^^17^^,^^19^^,^^20^ ARG1 deficiency prevalence calculations using allele frequencies in the general population with and without ACMG/AMP variant stratifications were subsequently performed (Figure 1). Allele frequencies (total number of variants/ total alleles in database) from gnomAD were incorporated into the Hardy-Weinberg equation to calculate ARG1 deficiency prevalence.

### ARG1 deficiency birth prevalence calculations

Seven different approaches to birth prevalence calculations were used, Models A to G.

Regarding model A, 73 published *ARG1* variants have been associated with ARG1 deficiency in the clinical scientific literature.^1^^,^^5^^,^^6^^,^^26^^,^^31^, ^32^, ^33^, ^34^, ^35^, ^36^, ^37^, ^38^, ^39^, ^40^, ^41^, ^42^ Only *ARG1* variants that have been published in peer-reviewed publications and abstracts were included. In many cases, patients were described with a clinical phenotype that correlated with Arg 1 deficiency, and this was attributed to 2 variants identified in the ARG1 gene. Further research was not always conducted to either confirm that both variants are in trans or that variants do cause arginase enzyme deficiency in in vitro experiments. Thus, assumptions have been made that the 2 variants identified in *ARG1* in these patients are pathogenic. These published *ARG1* variants (*n* = 73) were used in the first round of calculations (model A). Allele frequencies from gnomAD v2.1.1 for each of the 73 published *ARG1* variants were used. In the case of *ARG1* variants that were not present in the gnomAD database and were not located within the coding sequence or within 10 base pairs of an intron, the allele frequency was marked as 0/282,912 alleles (2 × [125,748 genomes + 15,708 genomes]). For *ARG1* variants that were not listed (among the 282,912 variants) in gnomAD during the present study, an assumed allele frequency of 1/300,000 (as an upper limit) was adopted for the calculations (Supplemental Table 1).

Regarding models B and C, 528 unpublished *ARG1* variants from gnomAD v2.1.1 were used for in silico guided birth prevalence estimations. Variants associated with 5′ untranslated region (UTR), introns, noncoding transcripts or 3′UTRs and synonymous *ARG1* variants were then excluded from this data set. In addition, 73 published *ARG1* variants (Supplemental Table 1) were also excluded from the in silico analysis, but allele frequencies (if available) of these variants were included in the calculations as for model A. For the remaining variants, each was analyzed with 3 in silico prediction tools: Sorting Intolerant From Tolerant,^43^ MutationTaster2,^44^ and PolyPhen 2.^45^ Variants that alter the protein coding sequence (frameshifts, deletions, insertions, duplications, etc) that cannot be analyzed by these in silico methods were deemed to be pathogenic. SIFT predictions were listed as “deleterious” or “tolerated”; PolyPhen2 predictions were listed as “benign,” “possibly damaging,” or “probably damaging”; and MutationTaster2 predictions were listed as “disease-causing” or “polymorphism” (Supplemental Table 1).

Regarding models D to F, ACMG/AMP variant classification criteria was applied to 73 published (Supplemental Table 1) and 302 unpublished *ARG1* variants from gnomAD v3.1.2 (Supplemental Table 2). Drawing upon the ACMG/AMP variant classifications, 3 further levels of prevalence prediction were conducted (models D, E, and F). For these analyses, allele frequencies for intronic variants deemed as splice variants were included if available. For *ARG1* variants that were not listed in gnomAD during the present study, an assumed allele frequency of 1/300,000 (as an upper limit) was adopted for the calculations (Supplemental Table 2).

Regarding model G, in recent years, further in silico prediction programs have been evaluated and are thought to be more accurate than the ones used for models B and C.^46^ We evaluated the data with 4 of these programs: BayesDel,^47^ MutPred2,^48^ REVEL,^49^ and VEST4.^50^ REVEL and BayesDel are meta-predictors, using prediction scores from multiple tools.

We repeated an analysis similar to model B, identifying a subgroup of *ARG1* variants from the 302 unpublished *ARG1* variants from gnomAD v3.1.2, which were consistently predicted as pathogenic/damaging by all 4 in silico prediction programs. Similar to that in prevalence calculations for models B and C, these gnomAD allele frequencies were combined with allele frequency data for the 73 published *ARG1* variants. Pathogenicity was denoted as suggested in Pejaver et al^46^: BayesDel score >0.13 = pathogenic (PP3), MutPred2 score >0.737 = pathogenic (PP3), REVEL score > 0.644 = pathogenic (PP3), and VEST4 score >0.764 = pathogenic (PP3). Again, as with models B and C, variants that alter the protein coding sequence (frameshifts, deletions, insertions, duplications, etc) that cannot be analyzed by these in silico methods were deemed to be pathogenic. Variants associated with 5′UTR, introns, noncoding transcripts or 3′UTRs, and synonymous *ARG1* variants were also excluded from this data set. For *ARG1* variants that were not listed in gnomAD during the present study, an assumed allele frequency of 1/300,000 (as an upper limit) was adopted for the calculations (Supplemental Table 2).

### Hardy-Weinberg relationship

In recessive disorders, the frequency of the disease (incidence/birth prevalence) corresponds to the frequency of the homozygous state for the disease allele, which is q^2^ according to the Hardy-Weinberg relationship (p^2^ + 2pq + q^2^ = 1, where the frequency of unaffected [AA] = p^2^, frequency of heterozygotes [Aa] = 2pq [carrier frequency], frequency of affected [aa] = q^2^ [birth prevalence]).

# variant alleles/ # total alleles = mutated allele frequency = q

# variant alleles/ # individuals = carrier rate

Allele count and total allele numbers were normalized to equal a population of 100,000 alleles. All variants deemed to be potentially “disease causing” in the models were totaled and incorporated into Hardy-Weinberg equation to calculate birth prevalence and carrier rate. The frequency of the disease corresponds to the combined frequency of the homozygous and compound heterozygous states; however, for the calculations used here, the minor allele q represents the estimated summed frequency of all pathogenic sequence variants in the gene.

### Summary of approaches used for calculation of birth prevalence and carrier rate for ARG1 deficiency

Seven levels of incidence prediction were conducted (Table 1).

**Table 1:** Summary of the models A to G

Models	Assumption	Projected Frequency of Cases
A	A total of 73 published *ARG1* variants are the main cause of arginase deficiency.	1 in 2,687,450
B	A total of 144 *ARG1* variants are the main cause of arginase deficiency. This comprises 73 published *ARG1* variants combined with 71 *ARG1* unpublished variants that were all predicted as pathogenic by 3 in silico prediction programs (SIFT, MutationTaster2, and PolyPhen2). All loss of function (frameshifts, nonsense, etc) and copy-number (deletions, duplications, etc) *ARG1* variants were included.	1 in 650,364
C	A total of 172 *ARG1* variants are the main cause of arginase deficiency. This comprises 73 published *ARG1* variants combined with 99 *ARG1* unpublished variants, which were predicted as pathogenic by 2 of 3 or 3 of 3 in silico prediction programs (SIFT, MutationTaster2, and PolyPhen2). All loss of function (frameshifts, nonsense, etc) and copy-number (deletions, duplications, etc) *ARG1* variants were included.	1 in 197,531
D	A total of 19 *ARG1* variants (16 published and 3 unpublished) are the main cause of arginase deficiency. These are the only *ARG1* variants that were classified as “pathogenic” by ACMG/AMP framework.	1 in 34,602,076
E	A total of 77 *ARG1* variants (46 published and 31 unpublished) are the main cause of arginase deficiency. These include variants that were classified as “likely pathogenic” or “pathogenic” by the ACMG/AMP framework.	1 in 5,948,840
F	A total of 242 *ARG1* variants (73 published, 169 unpublished) are the main cause of arginase deficiency. These include all variants that were classified as “pathogenic,” “likely pathogenic,” and “VUS” by the ACMG/AMP framework.	1 in 141,331
G	A total of 119 *ARG1* variants are the main cause of arginase deficiency. This comprises 73 published *ARG1* variants combined with 46 *ARG1* variants that were all predicted as pathogenic by 4 in silico prediction programs: BayesDel, MutPred2, REVEL, and VEST4. All loss of function (frameshifts, nonsense, etc) and copy-number (deletions, duplications, etc) *ARG1* variants were included.	1 in 1,562,500

*ACMG*, American College of Medical Genetics and Genomics; *AMP*, Association for Molecular Pathology.

#### Model A

This model assumes a very conservative estimation of birth prevalence focusing on published *ARG1* variants only. For this calculation, gnomAD allele frequency data for the 73 published *ARG1* variants was used.

#### Model B

This model is less conservative (relative to model A), using gnomAD allele frequency data for the 73 published *ARG1* variants combined with a selected subgroup of *ARG1* variants (*n* = 71) that were consistently predicted as pathogenic by 3 in silico prediction programs (ie, SIFT = “deleterious,” MutationTaster2 = “deleterious,” and PolyPhen2 = “probably damaging”). All loss of function (frameshifts, nonsense, etc) and copy-number (deletions, duplications, etc) *ARG1* variants were also included in the calculations as “deemed pathogenic.”

#### Model C

As for model B, except that variants were deemed as pathogenic if either 2 of 3 or 3 of 3 in silico prediction programs predicted variation as pathogenic (Polyphen2 prediction could be “possibly” or “probably damaging”) (*n* = 99). Model C represents the least conservative in silico guided estimation of ARG1 deficiency birth prevalence in this study.

#### Model D

Models D, E, and F incorporate the ACMG/AMP variant classification criteria. Model D assumes a conservative estimation of birth prevalence using only variants classified as P by ACMG/AMP (total *n* = 19 [*n* = 16 published, 3 unpublished]) for estimating ARG1 deficiency birth prevalence.

#### Model E

Akin to model D except that those variants classified as LP using ACMG/AMP criteria (total *n* = 58, [*n* = 30 published, 28 unpublished]) were also included for estimating ARG1 deficiency birth prevalence. The total number of P and LP variants included in calculations for model E = 77. Variants present in upstream genes, 5′UTR, noncoding transcripts, 3′UTRs, or synonymous changes were excluded from the calculation; however, allele frequencies for intronic splice site variants that classified as either P or LP were included in the calculations for model E.

#### Model F

This model assumes a very liberal estimation of birth prevalence by including ACMG/AMP P, LP, VUS for estimating ARG1 deficiency birth prevalence (total *n* = 242, [*n* = 73 published, 169 unpublished]). Variants present in upstream genes, 5′UTR, noncoding transcripts, 3′UTRs, or synonymous changes were excluded from the calculation; however, allele frequencies for intronic splice site variants that classified as either P or LP were included in the calculations for model F.

#### Model G

In recent years, further in silico prediction programs have been evaluated and are thought to be more accurate.^46^ We repeated an analysis similar to model B, using gnomAD allele frequency data for the 73 published *ARG1* variants, combined with a selected subgroup of *ARG1* variants (*n* = 46) that were consistently predicted as P by 4 in silico prediction programs: BayesDel, MutPred2, REVEL, and VEST4.

Hardy-Weinberg calculations for models A to G are detailed in Supplemental Table 3.

## Results

### ACMG/AMP classification of *ARG1* variants

**Figure 2:**
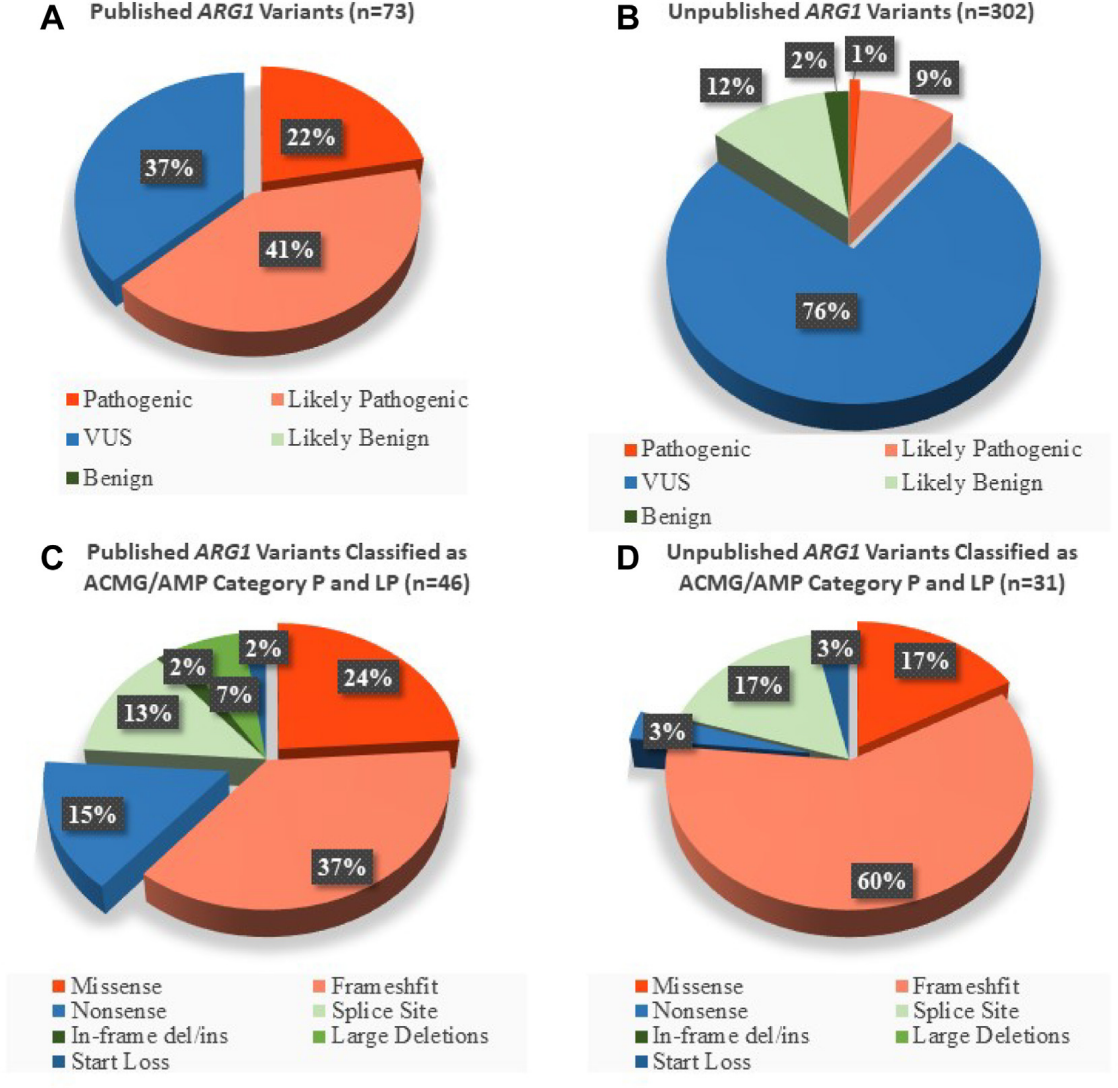
**Distribution of *ARG1* variants (*n* = 375) across American College of Medical Genetics and Genomics/Association for Molecular Pathology (ACMG/AMP) variant categories.** Pie chart representing the percentage of published *ARG1* variants (*n* = 73 variants) (A) and unpublished (*n* = 302 variants) *ARG1* variants (B) that were classified as pathogenic (P), likely pathogenic (LP), variant of uncertain significance (VUS), likely benign (LB), and benign (B). Pie chart showing the frequencies of different variant types (missense, frameshift, nonsense, splice-site, etc) that were classified as P and LP in the published (46 of 73 variants) *ARG1* variants (C) and unpublished *ARG1* variants (31 of 302) *ARG1* variants (D) studied.

Using ACMG/AMP variant classification approach, 16 of the 73 published *ARG1* variants were classified as P, 30 were classified as LP, and 27 as VUS (Table 2, Figure 2A). The 46 published *ARG1* variants that were classified as P or LP (combined, Figure 2C) included 17 frameshift, 11 missense, 7 nonsense, 6 splicing, 3 large deletions, 1 start loss, and 1 in-frame deletion variant(s). Of the 302 unpublished *ARG1* variants from gnomAD, we identified 3 variants that fulfilled ACMG/AMP criteria for classification as P, 28 variants that can be classified as LP, 229 variants that are VUS, 35 variants that we classified as LB, and 7 variants as B (Table 3, Figure 2B). The 31 unpublished *ARG1* variants from gnomAD that were classified as P or LP (combined, Figure 2D) included 19 frameshift, 5 missense, 1 nonsense, 5 splicing, and 1 start loss variant(s). Overall, 77 *ARG1* variants (46 published, and 31 unpublished) were classified as P or LP.

**Table 2:** Published *ARG1* variants from gnomAD that were classified as pathogenic, likely pathogenic, and VUS using the ACMG/AMP criteria (*n* = 73)

*ARG1* Variants Mapped to Transcript NM_000045.4 HGVSc	HGVSp	Variant Type	References	ACMG/AMP Attributes	ACMG/AMP Classification
c.560+5G>A	p.?	Splice site	^1^	PP3	VUS
c.231C>A	p.(Ser77Arg)	Missense	^6^	PP2	VUS
c.221G>T	p.(Gly74Val)	Missense	^1^	PP2	VUS
c.212G>C	p.(Arg71Thr)	Missense	^31^	PP2	VUS
c.58-3C>G^a^	p.?	Splice Site	^27^	PP3	VUS
c.23T>A	p.(Ile8Lys)	Missense	^1^	PP2	VUS
c.798A>C	p.(Lys266Asn)	Missense	^27^	BS1, PP2	VUS
c.del116_188^b^	p.?	Gross deletion of *ARG1*	^24^	PVS1	Likely pathogenic
NC_000006.12:g.131576244_131586997del^c^	p.?	Gross deletion of *ARG1*	^1^	PVS1	Likely pathogenic
c.306-611T>C	p.?	Intronic variant	^1^	BP7	VUS
c.466-2A>G	p.?	Splice site	^1^	PVS1	Pathogenic
c.666-2A>G	p.?	Splice site	^27^	PVS1	Pathogenic
c.466-1G>C	p.?	Splice site	^1^^,^^30^	PVS1	Pathogenic
c.121_122insCTT	p.(Lys41delinsThrTer)	Nonsense	^38^	PVS1	Likely pathogenic
c.124G>T	p.(Glu42∗)	Nonsense	^31^	PVS1	Pathogenic
c.132del	p.()	Frameshift	^27^	PVS1	Likely pathogenic
c.2T>C	p.(Met1?)	Start loss	^27^	PS1, PVS1	Pathogenic
c.206A>T	p.(Asn69Ile)	Missense	^33^	PP2, PP3	VUS
c.223A>T	p.(Lys75∗)	Nonsense	^1^	PVS1	Likely pathogenic
c.232dup	p.(Glu78fs)	Frameshift	^1^	PVS1	Likely pathogenic
c.263_266del	p.(Lys88fs)	Frameshift	^1^	PVS1	Pathogenic
c.282C>G	p.(Ser94Arg)	Missense	^27^	PP2, PP3	VUS
c.292G>A	p.(Gly98Ser)	Missense	^1^	PP2, PP3	VUS
c.295G>A	p.(Gly99Arg)	Missense	^29^	PP2, PP3	VUS
c.298G>A	p.(Asp100Asn)	Missense	^31^	PP2, PP3	VUS
c.316G>C	p.()	Missense	^32^	PP3, PP2, PM1, PM5	Likely pathogenic
c.32T>C	p.(Ile11Thr)	Missense	^1^	PP2, PP3	VUS
c.34G>T	p.(Gly12∗)	Nonsense	^1^	PVS1	Likely pathogenic
c.365G>A	p.(Trp122∗)	Nonsense	^1^	PVS1	Pathogenic
c.374C>T	p.(Ala125Val)	Missense	^1^	PM1, PP2, PP3	Likely pathogenic
c.383A>G	p.(Asp128Gly)	Missense	^1^	PP2, PP3	VUS
c.386_388del	p.(Ile129del)	In-frame deletion	^1^	PM1, PM4	Likely pathogenic
c.401C>T	p.(Thr134Ile)	Missense	^1^	PM1, PP2, PP3	Likely pathogenic
c.413G>T	p.(Gly138Val)	Missense	^1^	PM1, PP2, PP3	Likely pathogenic
c.422A>T	p.(His141Leu)	Missense	^1^	PM1, PP2, PP3	Likely pathogenic
c.425G>A	p.(Gly142Glu)	Missense	^1^	PM1, PP2, PP3	Likely pathogenic
c.434T>A	p.(Val145Glu)	Missense	^36^	PM1, PP2, PP3,	Likely pathogenic
c.446T>C	p.(Leu149Pro)	Missense	^27^	PM1, PP2, PP3	Likely pathogenic
c.523del	p.(Val175fs)	Frameshift	^1^	PVS1	Likely pathogenic
c.53G>A	p.(Gly18Glu)	Missense	^1^	PP2, PP3	VUS
c.539G>C	p.(Arg180Thr)	Missense	^1^	PP2, PP3	VUS
c.551del	p.(Pro184fs)	Frameshift	^27^	PVS1	Likely pathogenic
c.603_604del	p.(Glu202fs)	Frameshift	^27^	PVS1	Pathogenic
c.61C>T	p.(Arg21∗)	Nonsense	^1^	PVS1	Pathogenic
c.646_649del	p.(Leu216fs)	Frameshift	^1^	PVS1	Pathogenic
c.647T>C	p.(Leu216Pro)	Missense	^34^	PP3, PP2, PM3	Likely pathogenic
c.673delA	p.(Arg225fs)	Frameshift	^1^	PVS1	Likely pathogenic
c.682C>G	p.(His228Asp)	Missense	^27^	PP2, PP3	VUS
c.695A>T	p.(Asp232Val)	Missense	^1^	PP2, PP3	VUS
c.700G>C	p.(Asp234His)	Missense	^1^	PP2, PP3	VUS
c.70del	p.(Val24fs)	Frameshift	^1^	PVS1	Likely pathogenic
c.703G>A	p.(Gly235Arg)	Missense	^1^	PS1, PP3, PP2	Likely pathogenic
c.703G>C	p.(Gly235Arg)	Missense	^1^	PS1, PP3, PP2	Likely pathogenic
c.704del	p.(Gly235fs)	Frameshift	^5^	PVS1	Likely pathogenic
c.707T>C	p.(Leu236Pro)	Missense	^28^	PP2, PP3	VUS
c.708_712dup	p.(Pro238fs)	Frameshift	^27^	PVS1	Likely pathogenic
c.78del	p.(Gly27fs)	Frameshift	^1^	PVS1	Pathogenic
c.80G>A	p.(Gly27Asp)	Missense	^1^	PP2, PP3	VUS
c.820G>A	p.(Asp274Asn)	Missense	^37^	PP2, PP3	VUS
c.837C>A	p.(Asn279Lys)	Missense	^33^	PP2, PP3	VUS
c.844del	p.(Leu282fs)	Frameshift	^1^	PVS1	Pathogenic
c.849del	p.(Lys284fs)	Frameshift	^27^	PVS1	Likely pathogenic
c.866dup	p.(Thr290fs)	Frameshift	^35^	PVS1	Likely pathogenic
c.871C>T	p.(Arg291∗)	Nonsense	^1^	PVS1	Pathogenic
c.877del	p.(Val293fs)	Frameshift	^27^	PVS1	Likely pathogenic
c.892G>C	p.(Ala298Pro)	Missense	^1^	PP2	VUS
c.913G>A	p.(Gly305Arg)	Missense	^1^	PP2, PP3	VUS
c.923G>A	p.(Arg308Gln)	Missense	^1^^,^^57^	PP2, PP3	VUS
c.93del	p.(Arg32fs)	Frameshift	^1^	PVS1	Pathogenic
Exon 1 Deletion	p.?	Exon deletion	^27^	PVS1	Likely pathogenic
c.130+1 G>A	p.?	Splice site	^27^	PVS1	Pathogenic
c.57+1G>A	p.?	Splice site	^1^	PVS1	Pathogenic
c.802+2T>G	p.?	Splice site	^27^	PVS1	Likely pathogenic

*ACMG*, American College of Medical Genetics and Genomics; *AMP*, Association for Molecular Pathology.

a Although c.58-3C>G is within a splice region, the PVS1 criteria was not applied because of recent recommendations.^16^

b The variant c.del116_188 involves a complex rearrangement resulting in gross deletion of *ARG1*.^24^

c The variant NC_000006.12:g.131576244_131586997del has a gross deletion of *ARG1* reportedly involving about 10,753 nucleotides possibly encompassing intron 1 to poly(A) site.^1^

**Table 3:** Unpublished *ARG1* variants from gnomAD that were classified as pathogenic, likely pathogenic, and VUS using the ACMG/AMP criteria (*n* = 31)

*ARG1* Transcript(s)	*ARG1* Variants Mapped to Transcript HGVSc	HGVSp	Variant Type	ClinVar Classification	ACMG/AMP Attributes	ACMG/AMP Classification
NM_000045.3	c.131-4_131-2del	p.?	Splice site	X	PVS1	Likely pathogenic
NM_000045.4	c.131-7_131-2del	p.?	Splice site	VUS	PVS1	Likely pathogenic
NM_001244438.1^a^	c.272delAins	p.(Lys91fs)	Frameshift	X, a similar variant NM_000045.4: c.272del (NP_000036.2: p.(Gly91fs)) was classified as pathogenic	PVS1	Likely pathogenic
	c.161dupA	p.(Asp54fs)	Frameshift	X	PVS1	Likely pathogenic
	c.145_146del	p.(Leu49fs)	Frameshift	VUS	PVS1	Likely pathogenic
	c.146dup	p.(Leu49fs)	Frameshift	VUS	PVS1	Likely pathogenic
	c.139_140del	p.(Asn47fs)	Frameshift	X	PVS1	Likely pathogenic
	c.129del	p.(Val44fs)	Frameshift	Pathogenic/likely pathogenic	PVS1	Pathogenic
	c.57+2_57+6del	p.?	Splice site	Likely pathogenic	PVS1	Likely pathogenic
	c.3G>A	p.(Met1?)	Start loss	Pathogenic/likely pathogenic	PS1, PVS1	Pathogenic
	c.627_628del	p.(Glu210fs)	Frameshift	Pathogenic	PVS1	Likely pathogenic
	c.938del	p.(Gly313fs)	Frameshift	X	PVS1	Likely pathogenic
	c.946C>T	p.(Arg316Trp)	Missense	VUS	PM5, PP2, PP3	Likely pathogenic
	c.164_165del	p.(Val55fs)	Frameshift	Likely pathogenic	PVS1	Likely pathogenic
	c.146delTins	p.(Leu49fs)	Frameshift	X	PVS1	Likely pathogenic
	c.169_170del	p.(Asp57fs)	Frameshift	X	PVS1	Likely pathogenic
	c.161delAinsNN	p.(Asp54fs)	Frameshift	X	PVS1	Likely pathogenic
	c.320dupG	p.(Asp108fs)	Frameshift	X	PVS1	Likely pathogenic
	c.340G>A	p.(Gly114Arg)	Missense	X	PP3, PP2, PM1, PM5	Likely pathogenic
	c.394G>T	p.(Asp132Tyr)	Missense	Likely pathogenic	PP3, PP2, PM1	Likely pathogenic
	c.573_576del	p.(Asp191fs)	Frameshift	X	PVS1	Likely pathogenic
	c.950_953del	p.(Glu317fs)	Frameshift	X	PVS1	Likely pathogenic
	c.962del	p.(Lys321fs)	Frameshift	X	PVS1	Pathogenic
	c.395A>G	p.(Asp132Gly)	Missense	X	PP3, PP2, PM1, PM5	Likely pathogenic
	c.437G>T	p.(Gly146Val)	Missense	Pathogenic/VUS	PP3, PP2, PM1	Likely pathogenic
	c.489+1G>A	p.?	Splice site	X	PVS1	Likely pathogenic
	c.549_552del	p.(Tyr184fs)	Frameshift	X	PVS1	Likely pathogenic
	c.620C>A	p.(Ser207∗)	Nonsense	X	PVS1	Likely pathogenic
	c.925_928del	p.(Ala310fs)	Frameshift	X	PVS1	Likely pathogenic
	c.703delAins	p.(Ile235fs)	Frameshift	X	PVS1	Likely pathogenic
	c.827-2A>T	p.?	Splice site	X	PVS1	Likely pathogenic

X implies that the variant was not reported on ClinVar at the time of review.

*ACMG*, American College of Medical Genetics and Genomics; *AMP*, Association for Molecular Pathology; *VUS*, variant of uncertain significance.

a Variants were also mapped to the newer transcript NM_001244438.2 and to the MANE select transcript NM_000045.4.

**Figure 3:**
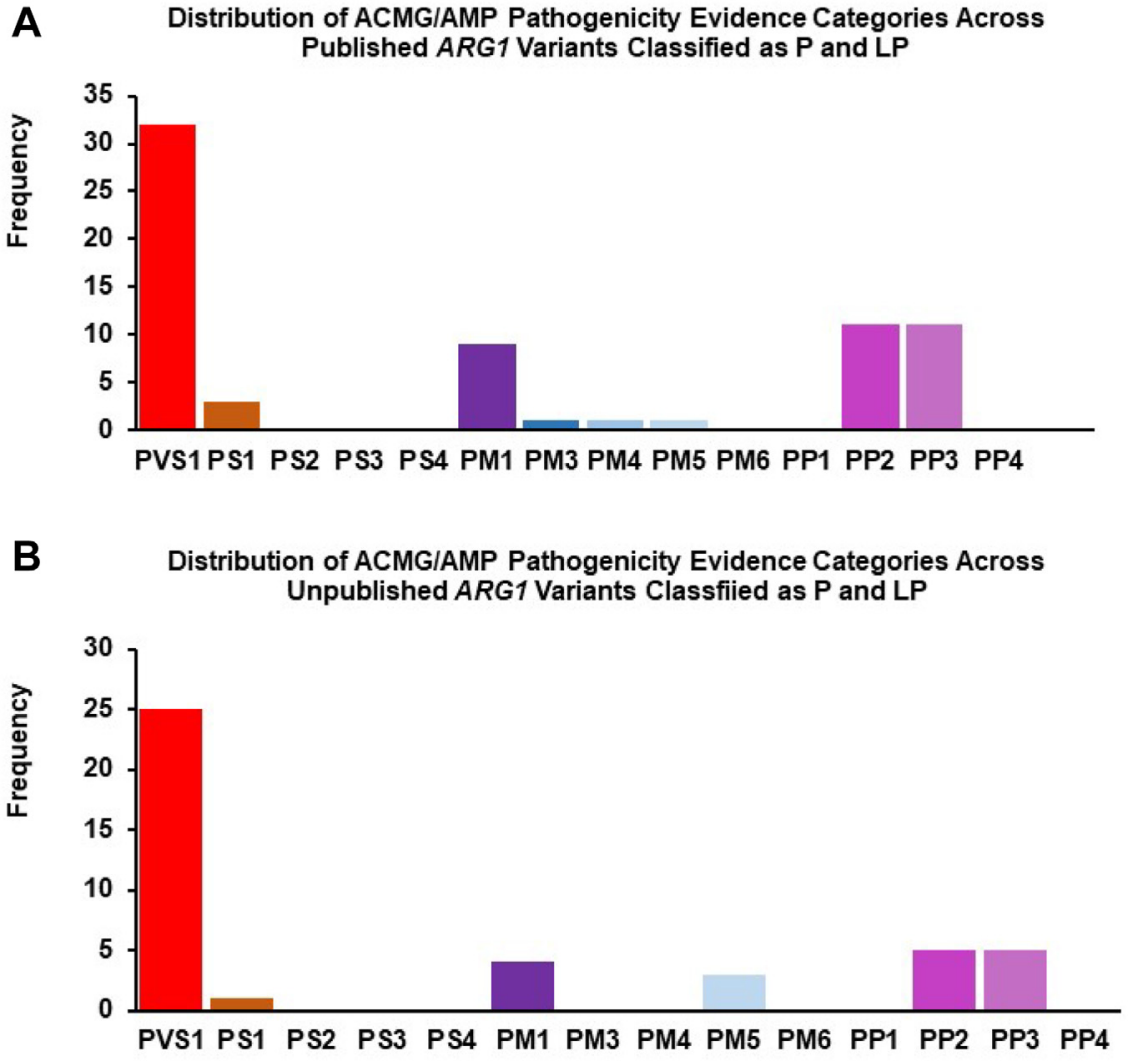
**Distribution of American College of Medical Genetics and Genomics/Association for Molecular Pathology (ACMG/AMP) pathogenicity evidence categories (very strong [PVS1], strong [PS1-4], moderate [PM1-6], supporting [PP1-4]) across *ARG1* variants that were classified as pathogenic (P), and likely pathogenic (LP) in this study.** A. Published (*n* = 46 variants). B. Unpublished (*n* = 31 variants).

Of the 16 pathogenicity evidence categories in the ACMG/AMP variant classification framework, PVS1 was most frequently used for both published (Table 2, Figure 3A) and unpublished (Table 3, Figure 3B) *ARG1* variants that were classified as P or LP. This was followed by PP2 and PP3. For all published *ARG1* variants that were classified as P or LP, ACMG/AMP pathogenicity evidence categories that were not applied include PS2, PS3, PS4, PM2, PM6, PP1, PP4, and PP5. In addition to the aforementioned, the ACMG/AMP pathogenicity evidence categories PM3 and PM4 were not applied to any of the 31 unpublished *ARG1* variants from gnomAD that were classified as P or LP. Of these 31 unpublished *ARG1* variants that we classified as P or LP based on ACMG/AMP variant assessment framework, 20 were absent from ClinVar, 1 variant was classified as P and 3 as LP on ClinVar, and 4 were classified as VUS, whereas 3 had conflicting classifications on ClinVar. Of these 3 variants with conflicting classifications on ClinVar, 2 were classified as both P and LP, whereas 1 was classified as both P and VUS.

### Birth prevalence and carrier rate calculations for ARG1 deficiency

Using gnomAD allele frequency of published *ARG1* variants (*n* = 73) only (model A), mutant allele frequency was estimated at 61 per 100,000 (see Supplemental Tables 1 and 3), carrier frequency was 1 in 820, and birth prevalence was **1 in 2,687,450 (model A)**. For model B, gnomAD allele frequency data for the 73 published *ARG1* variants and the unpublished *ARG1* variants (total *n* = 71) that were consistently predicted as pathogenic by 3 in silico prediction programs were combined, and the mutant allele frequency was estimated at 124 per 100,000. Carrier frequency was 1 in 404, and birth prevalence was **1 in 650,364 (model B)**. For model C, which combined gnomAD allele frequency data for the 73 published *ARG1* variants and unpublished *ARG1* variants were deemed as pathogenic by at least 2 of 3 in silico prediction programs, mutant allele frequency was estimated at 225 per 100,000, carrier frequency was 1 in 223, and birth prevalence was **1 in 197,531 (model C)**.

Applying the gnomAD allele frequency data for only *ARG1* variants that were classified as P by the ACMG/AMP criteria (*n* = 16 published, 3 unpublished, model D), mutant allele frequency was estimated at 17 per 100,000, carrier frequency of 1 in 2942, and birth prevalence of **1 in 34,602,076 (model D)**. Using the gnomAD allele frequency of only *ARG1* variants that were classified by the ACMG/AMP criteria as either LP and P (total *n* = 77, *n* = 46 published, 31 unpublished, model E), mutant allele frequency was estimated at 41 per 100,000, carrier frequency was 1 in 1220, and birth prevalence was **1 in 5,948,840 (model E)**. Incorporating the gnomAD allele frequency of *ARG1* variants that were classified by the ACMG/AMP criteria as either VUS, LP, and P (total *n* = 242; *n* = 73 published, 169 unpublished, model F), mutant allele frequency was estimated at 266 per 100,000, carrier frequency was 1 in 188, and birth prevalence was **1 in 141,331 (model F)**.

The 3 in silico prediction tools used for models B and C are now known to be less accurate than more recent meta-predictors. ACMG-compliant calibrations of a number of these computational tools have been published^46^ (PMID: 36413997). We compared the use of 4 of these tools (BayesDel, MutPred2, REVEL, and VEST4) with the results from more commonly used in silico prediction programs (SIFT, MutationTaster2, and PolyPhen2).

For model G, gnomAD allele frequency data for the 73 published *ARG1* variants and the unpublished *ARG1* variants (total *n* = 46) that were consistently predicted as pathogenic by 4 in silico prediction programs were combined, and the mutant allele frequency was estimated at 80 per 100,000. Carrier frequency was 1 in 626, and birth prevalence was **1 in 1,562,500 (model G)**. These data should be compared with model B, in which similar data were analyzed by the older in silico tools.

## Discussion

Estimating the global birth prevalence of ARG1 deficiency based on available evidence base is a challenging but imperative step toward addressing the unmet health needs and for improving patient care.^8^^,^^9^^,^^11^ In this study, we adopted the ACMG/AMP variant stratifications and various in silico prediction models for the selection of *ARG1* mutant allele frequencies incorporated into calculations for estimating global birth prevalence of ARG1 deficiency. Using 7 different models (models A-G), our estimates of total mutant allele frequencies ranged from 17 to 226 per 100,000 with carrier frequency range of 1 in 188 to 2942 and birth prevalence approximations ranging from 1 in 141,331 to 34,602,076. A recent study by Catsburg et al^11^ estimated the global birth prevalence of ARG1 deficiency to be about 1 in 357,000 using allele frequencies of 28 published *ARG1* variants across 38 countries. Although Catsburg et al^11^ estimates are in accord with the calculations from our most liberal models (model F and model C), our findings provide more comprehensive estimates that encompass a diverse range of birth prevalence possibilities (models A-G, involving 375 *ARG1* variants) considering the growing *ARG1* variant numbers in genetic databases.

Data set from NBS in the United States and the Urea Cycle Disorders Consortium suggested that the birth prevalence of ARG1 deficiency is about 1 in 1,000,000 live births.^8^ ARG1 deficiency birth prevalence was estimated at 1:2,000,000 in Japan.^51^ In Massachusetts, United States, approximately 1 positive case was identified in about 200,000 newborn screens.^52^ Therrell et al^9^ estimated the birth prevalence of ARG1 deficiency as 1 in 119,500 based on data from all US states that screened newborns for hyperargininemia between 1999 and 2015. Overall, the birth prevalence of ARG1 deficiency has been estimated hitherto to be between 1:350,000 and 1:1,000,000.^53^ The current study provides broad estimates from a variable range of conservative, and liberal evidence-based models (models A-G). Of these estimates, model B (birth prevalence of 1 in 650,364), model C (birth prevalence of 1 in 197,531), and model F (birth prevalence of 1 in 141,331) are more closely aligned with the data set from previous studies.^8^^,^^9^^,^^11^ The unifying premise for calculations models B, C, and F relative to models A, D, and E is an increased assignment of pathogenicity to many *ARG1* variants that currently fall into the VUS category of the ACMG/AMP framework. These findings underscore the need for an improved understanding of *ARG1* variants that currently fall into VUS category.

For this study, we also took advantage of the development of newer in silico prediction tools to compare their assessment of pathogenicity in our data set of *ARG1* variants. Although in silico predictors should not be used alone to classify the pathogenicity of a variant (as reviewed in Pejaver et al^46^), we chose to combine results from 3 more commonly used prediction tools (SIFT, MutationTaster2, and PolyPhen2) and compare them with results from more recently developed tools (BayesDel, MutPred2, REVEL, and VEST4). Data were analyzed at different time points (model B analyzed 528 unpublished *ARG1* variants from gnomAD v2.1.1, and model G analyzed 302 unpublished *ARG1* variants from gnomAD v3.1.2), but the overall data yield should be comparable. Both data sets included allele frequencies for variants that were unable to be analyzed by these methods but were deemed pathogenic (eg, loss of function and copy-number variants), as well as 73 *ARG1* variants that were published and thus also deemed pathogenic.

Less allele frequency data were available for *ARG1* variants deemed pathogenic by all 4 of the newer in silico prediction programs, resulting in a smaller total allele frequency and lower resultant birth prevalence (1:1,562,500 for model G vs 1:650,364 for model B). Although the data set of *ARG1* variants used for model B was larger, this is likely because of more stringent assignments of pathogenicity to variants by more recently developed programs.

We found that a large proportion of *ARG1* variants that are present in genetic databases hitherto lack adequate evidence of pathogenicity (Figure 2A, B; Table 2 and Supplemental Table 2), thus highlighting a growing need for functional characterization studies and an increased utilization of available clinical data to guide *ARG1* variant classifications and precise estimation of ARG1 deficiency birth prevalence. It is likely that the majority of these variants are benign, but if they remain unclassified as VUS, it can be very hard to give patients the information they need toward providing a diagnosis (or not) of arginase deficiency. The ACMG/AMP 2015 guidelines have been a significant step toward a unified classification of gene variants for many monogenic disorders.^15^^,^^54^ The ACMG/AMP guidelines are increasingly being refined with growing applications across Mendelian disorders.^16^, ^17^, ^18^, ^19^, ^20^, ^21^, ^22^^,^^55^ Our observations that PVS1 was the most frequently used of the ACMG/AMP pathogenicity evidence categories for variants that classified as LP or P suggest the need for targeted studies that will yield more data set for the other ACMG/AMP pathogenicity evidence categories. We found that frameshift and missense mutations accounted for the largest proportion of *ARG1* variants that classified as LP and P. Although the ACMG/AMP guidelines are overwhelmingly applied across clinical laboratories and are aimed at promoting consistent variant interpretation,^54^ differences in their applications due to subjective interpretation of some criteria have triggered follow-up suggestions.^16^^,^^17^^,^^19^ Our findings that conflicting classifications of some *ARG1* variants exist on ClinVar, with some laboratories citing the same ACMG/AMP guidelines, suggest that ARG1 gene-specific modifications to ACMG/AMP guidelines may be beneficial for harmonized classifications similar to some other genes.^21^^,^^55^, ^56^, ^57^ With the growing discussions of reproductive carrier screening for inborn errors of metabolism,^58^, ^59^, ^60^ functional characterization studies and the application of the growing clinical data to facilitate re-classifications of *ARG1* VUS are increasingly becoming crucial. It is hoped that an improved cross-communication between biochemical and molecular laboratories will also pave the way for enhanced reclassifications of *ARG1* VUS.

## Data Availability

Any data not included as supplemental material are available on request.

## Conflict of Interest

Andreas Schulze is a paid consultant and member of the advisory boards for Aeglea Biotherapeutics, HZNP Canada Ltd, ModernaTX Inc, and Recordati Rare Diseases Canada Inc. Andreas Schulze is site principal investigator on clinical trials sponsored by ModernaTX Inc and 10.13039/100013220Ultragenyx Canada Inc. All other authors declare no conflicts of interest.

## Additional Information

Supplemental Table 1 gnomAD v2.1.1 *ARG1* published variants (n=73) used for carrier frequency and birth prevalence calculations.

Supplemental Table 2 gnomAD 3.1.2 *ARG1* variants classification using the ACMG/AMP criteria.

Supplemental Table 3 Calculations for Models A – F.
