# Supplementary material for: ACMG/AMP variant classification framework in arginase 1 deficiency: Implications for birth prevalence estimates and diagnostics
Source: Genet Med Open. 2024 Jan 23;2:101815. doi: 10.1016/j.gimo.2024.101815 (PMC11613747; doi:10.1016/j.gimo.2024.101815)
Supplement: Supplemental Table 3 — Calculations for Models A – F. [file mmc3.pdf]

**Supplementary Table 3:** Calculations for Models A – F.

**Model A (all published ARG1 variants)**

| | | | | | |
|---|---|---|---|---|---|
| Mutated allele frequency = 61/100,000 = q | q | 0.00061 | | | |
| Frequency of homozygous genotype (aa) = $q^2$ | $q^2$ | 3.721E-07 | $1/q^2$ | **Birth prevalence = 1:** | **2,687,450** |
| p + q = 1, therefore p= 1-q | 1-q | 0.99939 | | | |
| Carrier rate = 2pq | 2pq | 0.0012193 | | | |
| | 1/2pq | 820.17244 | | **Carrier rate = 1:** | **820** |

**Model B (All unpublished ARG1 variants predicted as Pathogenic by 3 in silico methods + mutated allele freq data for published ARG1 variants)**

| | | | | | |
|---|---|---|---|---|---|
| Mutated allele frequency = 124/100,000 = q | q | 0.00124 | | | |
| Frequency of homozygous genotype (aa) = $q^2$ | $q^2$ | 1.538E-06 | $1/q^2$ | **Birth prevalence = 1:** | **650,364** |
| p + q = 1, therefore p= 1-q | 1-q | 0.99876 | | | |
| Carrier rate = 2pq | 2pq | 0.0024769 | | | |
| | 1/2pq | 403.72643 | **Carrier rat** | **Carrier rate = 1:** | **404** |

**Model C (All unpublished ARG1 variants predicted as Pathogenic by at least 2/3 in silico methods + mutated allele freq data for published ARG1 variants)**

| | | | | | |
|---|---|---|---|---|---|
| Mutated allele frequency = 225/100,000 = q | q | 0.00225 | | | |
| Frequency of homozygous genotype (aa) = $q^2$ | $q^2$ | 5.063E-06 | $1/q^2$ | **Birth prevalence = 1:** | **197,531** |
| p + q = 1, therefore p= 1-q | 1-q | 0.99775 | | | |
| Carrier rate = 2pq | 2pq | 0.0044899 | | | |
| | 1/2pq | 222.72335 | **Carrier rat** | **Carrier rate = 1:** | **223** |

**Model D (All unpublished ARG1 variants classified as Pathogenic by ACMG criteria, including ARG1 published variants)**

| | | | | | |
|---|---|---|---|---|---|
| Mutated allele frequency = 17/100,000 = q | q | 0.00017 | | | |
| Frequency of homozygous genotype (aa) = $q^2$ | $q^2$ | 2.89E-08 | $1/q^2$ | **Birth prevalence = 1:** | **34,602,076** |
| p + q = 1, therefore p= 1-q | 1-q | 0.99983 | | | |
| Carrier rate = 2pq | 2pq | 0.0003399 | | | |
| | 1/2pq | 2941.6766 | **Carrier rat** | **Carrier rate = 1:** | **2,942** |

**Model E (All unpublished ARG1 variants classified as Pathogenic or Likely Pathogenic by ACMG criteria, including ARG1 published variants)**

| | | | | | |
|---|---|---|---|---|---|
| Mutated allele frequency = 41/100,000 = q | q | 0.00041 | | | |
| Frequency of homozygous genotype (aa) = $q^2$ | $q^2$ | 1.681E-07 | $1/q^2$ | **Birth prevalence = 1:** | **5,948,840** |
| p + q = 1, therefore p= 1-q | 1-q | 0.99959 | | | |
| Carrier rate = 2pq | 2pq | 0.0008197 | | | |
| | 1/2pq | 1220.0124 | **Carrier rat** | **Carrier rate = 1:** | **1,220** |

**Model F (All unpublished ARG1 variants classified as Pathogenic, Likely Pathogenic or VUS by ACMG criteria, including ARG1 published variants)**

| | | | | | |
|---|---|---|---|---|---|
| Mutated allele frequency = 266/100,000 = q | q | 0.00266 | | | |
| Frequency of homozygous genotype (aa) = $q^2$ | $q^2$ | 7.076E-06 | $1/q^2$ | **Birth prevalence = 1:** | **141,331** |
| p + q = 1, therefore p= 1-q | 1-q | 0.99734 | | | |
| Carrier rate = 2pq | 2pq | 0.0053058 | | | |
| | 1/2pq | 188.47126 | **Carrier rat** | **Carrier rate = 1:** | **188** |

**Model G (All unpublished ARG1 variants predicted as Pathogenic by 4 meta-predictor in silico methods + mutated allele freq data for published ARG1 variants)**

| | | | | | |
|---|---|---|---|---|---|
| Mutated allele frequency = 80/100,000 = q | q | 0.0008 | | | |
| Frequency of homozygous genotype (aa) = $q^2$ | $q^2$ | 6.4E-07 | $1/q^2$ | **Birth prevalence = 1:** | **1,562,500** |
| p + q = 1, therefore p= 1-q | 1-q | 0.9992 | | | |
| Carrier rate = 2pq | 2pq | 0.0015987 | | | |
| | 1/2pq | 625.5004 | **Carrier rat** | **Carrier rate = 1:** | **626** |
